# Psoriasis and Its Relationship With Somatosensory Amplification, Health Anxiety, and Depression

**DOI:** 10.7759/cureus.34037

**Published:** 2023-01-21

**Authors:** Gülhan Gürel, Işın Öncü, Dilara Güler, İrem Nur Durusu Türkoğlu, Seçil Soylu

**Affiliations:** 1 Department of Dermatology, Faculty of Medicine, Afyonkarahisar Health Sciences University, Afyonkarahisar, TUR; 2 Department of Dermatology, Kadirli State Hospital, Osmaniye, TUR; 3 Department of Dermatology, Meram State Hospital, Konya, TUR

**Keywords:** psoriasis, somatosensory amplification, health anxiety, depression, anxiety

## Abstract

Objective

The present study investigates the relationship between psoriasis and diseases such as health anxiety, depression, and somatosensory amplification.

Methods

The participating patients (n=117, including 60 psoriasis patients and 57 controls) filled out the Beck Depression Inventory (BDI), Beck Anxiety Inventory (BAI), Somatosensory Amplification Scale (SSAS), and Health Anxiety Inventory (HAI) questionnaires.

Results

The mean scores from SSAS, HAI, and BDI were significantly higher in the psoriasis group than in the control group (p<0.05 in all comparisons). When the group medians of BAI were evaluated, the differences were not statistically significant, although BAI medians were higher in the patient group. Furthermore, a moderate correlation was found between the involvement of specific areas (especially the scalp and face) and SSAS scores.

Conclusion

Patients with psoriasis score highly in depression, health anxiety, and somatosensory amplification, and there was a moderate correlation between specific body area involvement (especially the scalp and face) and SSAS score. The results of this study seem to indicate that psychiatric assessment and treatment approaches should be included in the treatment of such chronic skin diseases as psoriasis that follow a life-long remission and relapse pattern.

## Introduction

Psoriasis is a common chronic inflammatory skin disease that affects approximately 2-3% of the global population [1]. It has different clinical phenotypes, but the most frequent is chronic plaque psoriasis, which is characterized by erythematous plaque and scaling. Lesions typically affect the scalp, knees, elbows, and sacral region, although nail and joint involvement may also be present [2]. Psoriasis is caused by a combination of genetic and environmental factors. Recent immunological studies have emphasized the key role of cytokines, especially IL-17 and IL-23, in epidermal hyperproliferation, and the role of Th17, TNFα, and interferons [1,3].

The various proinflammatory interleukins involved make psoriasis a multisystem disease that is associated with such comorbidities as psoriatic arthritis, coronary artery disease, diabetes, obesity, inflammatory bowel disease, and psychological problems [4,5]. Patients may also present with physiological stress, pain, stigmatization, embarrassment, itching, and physical discomfort, which can result in impaired social function productivity and physical activity and even sleep problems. Psoriasis is a disease that can have severe effects on mental health and quality of life, and several studies have been conducted in recent years investigating the effects of this condition on the general state of health [6]. The early detection of comorbid psychiatric disorders and the identification of contributing factors are important for the prognosis of the disease.

Somatosensory amplification, which was first described by Barsky, refers to a tendency to experience normal and non-noxious sensations such as intense and disturbing [7]. Health anxiety, in turn, refers to the belief that one has a serious medical condition based on the misinterpretation of bodily sensations and a high level of anxiety about health. Evidence suggests a close association between increased somatic symptom burden and health anxiety [8]. To the best of our knowledge, however, there has been no study to date assessing the Somatosensory Amplification Scale (SSA) and health anxiety in psoriasis patients. The present study, therefore, investigates whether SSA, health anxiety, depression, and anxiety symptoms differ between psoriasis patients and a healthy control group.

## Materials and methods

Study sample

Included in this cross-sectional case-control study were patients admitted to the dermatology outpatient clinic due to psoriasis between July 2020 and July 2021. The study was planned in accordance with the principles of the Declaration of Helsinki after the granting of local ethics committee approval (2011-KAEK-2 2020/270).

Those who agreed to participate in the study were informed about the study goals and written informed consent was obtained from all of the respondents. Included in the study were patients over the age of 18 years with the cognitive ability to complete the scales, and with no history of psychiatric disease, serious systemic disease, malignancy, or neurological disease. Patients on psychiatric drugs or those with neurological or psychiatric diseases were excluded from the study. A total of 60 patients who met the study criteria were included in the study while the control group comprised 57 volunteers with no physical or psychiatric disease. The sociodemographic data of all participants were recorded. The Psoriasis Area Severity Index (PASI) was used to determine psoriasis disease severity and the Nail Psoriasis Severity Index (NAPSI) was used to determine nail psoriasis disease severity. Disease parameters, such as duration of disease, presence of special area involvement (scalp, hand, face, nails, and genitals), presence of arthritis, family history, and previous treatments were also recorded for the psoriasis patients.

Scales used in the study

Somatosensory Amplification Scale (SSAS)

This 10-item scale was developed by Barsky et al. for the evaluation of the tendency to experience normal bodily sensations as disturbing [7]. The items are scored from 1-5 points, with most being related to a range of disturbing bodily sensations that are not symptoms of the disease. The total score on this scale indicates the level of exaggeration of bodily sensations. Gulec and Sayar performed a reliability and validity study for the Turkish population [9].

Health Anxiety Inventory (HAI) (Short Version)

The Health Anxiety Inventory is a self-administered scale that consists of 18 items. The first 14 items of the scale question the mental state of the patients while the remaining four items ask the patients to consider their possible mental state should they develop a critical illness. Each item is scored between 0-3, with a high score indicating a high level of health anxiety. The Turkish reliability and validity study was performed by Aydemir et al. [10].

Beck Depression Inventory (BDI)

Beck et al. developed this scale for the evaluation of depression-associated physical, emotional, cognitive, and motivational symptoms [11]. It is a self-reported scale comprising 21 questions with four available answers. Each answer is scored on a scale of 0-3 points (based on the intensity of the answer) and the total score ranges from 0 to 63 points. The Turkish reliability and validity study was performed by Hisli [12].

Beck Anxiety Inventory (BAI)

Beck et al. developed this scale for the evaluation of anxiety symptoms [13]. Similar to BDI, the BAI is also a self-administered inventory consisting of 21 questions scored between 0 and 3 points, with a total score ranging from 0-63 points. The higher the total score, the higher the severity of the individual’s anxiety. The Turkish reliability and validity study was performed by Ulusoy et al. [14].

Statistical analysis

All analyses were carried out in IBM SPSS Statistics (Version 20.0. Armonk, NY: IBM Corp.). Kolmogorov-Smirnov tests were applied to determine the normal distribution of the data, a Mann-Whitney U test was used to compare non-parametric variables, a student’s t-test was used for the comparison of continuous variables, and a chi-square test was used for the categorical variables. Pearson’s correlation analysis was applied for the examination of the correlations between disease severity, disease duration, gender, educational status, body mass index (BMI), and special area involvement, and the SSAS, HAI, and BDI scales scores for the examination of the psoriasis group. P-values of <0.05 were accepted as significant. The reliability coefficients of the study scales were calculated separately for each group, and each of the calculated Cronbach’s α values was greater than 0.600, suggesting that the scales could be considered reliable.

## Results

A comparison of certain demographic characteristics of the patient and control groups study revealed the mean age and female-to-male ratio to be similar in the two groups (p=0.593 and p=0.649 for each). Concerning marital status, there was no statistically significant difference in the respective rates between the two groups (p=0.430), and there was also no statistically significant difference in the level of education of the two groups (p=0.178). An assessment of occupations revealed no statistical difference between the groups (p=0.152). The patient and control groups did not differ statistically in terms of smoking and alcohol use (p=0.206 and p=0.516 for each). The BMI was slightly higher in the patient group, but there was no statistical difference (p=0.117).

The mean PASI value was 8.22 ± 8.95 and the mean NAPSI value was 8.56 ± 16.61 in the patient group, and the patients had a mean disease duration of 10 years. Some specific clinical parameters in psoriasis patients are presented in Table 1.

**Table 1:** Comparison of demographic and clinical parameters between the patient and control groups *: Student’s t-test; **: Chi-square test; PASI: Psoriasis Area Severity Index; NAPSI: Nail Psoriasis Severity Index

	Control (n=57)	Patient (n=60)	p-value
Age	40.75 ± 14.30	42.20 ± 14.85	0.593*
Gender (F/M)	28/29	32/28	0.649**
Marital status			
Married	40	47	0.430**
Single	15	10	
Widowed/Divorced	2	3	
Education level			
Primary school	17	29	0.178**
Secondary school	7	5	
High school	7	8	
Bachelor’s/Master’s/Doctoral	26	18	
Profession			
Student	6	5	0.152**
Civil Servant	16	7	
Worker	6	3	
Self-employed	8	12	
Housewife	15	20	
Unemployed/Pensioner	6	13	
Smoking			
Yes	13	20	0.206**
No	44	40	
Alcohol			
Yes	9	7	
No	48	53	0.516**
Body Mass Index (BMI)	26.76 ± 4.82	28.43 ± 6.43	0.117*
Duration of disease (years)	-	10	NA
PASI	-	8.22 ± 8.95	NA
NAPSI	-	8.56 ± 16.61	NA
Psoriatic arthritis (yes/no)	-	12/48	NA
Specific body area involvement			
Scalp	-	38	
Hands	-	16	
Face	-	13	
Nails	-	24	
Genitals	-	17	

The difference between the total mean SSAS scores of the patient and control groups was statistically significant (p=0.004), being significantly higher in the patient group than in the control group. There was a statistically significant difference in the median HAI scores of the patient and control groups (p<0.001), with the median HAI score being significantly higher in the patient group than in the control group.

The median BAI score was not statistically significantly different between the two groups (p=0.120), although the patient group had a higher median BAI score. The median BDI score, in turn, was statistically significantly different between the two groups (p=0.002), with the median BDI score being significantly higher in the patient group than in the healthy control group (Table 2).

**Table 2:** Comparison of the study scales between the patient and control groups *: The student’s t-test was used for independent groups. Descriptive statistics were expressed as mean ± standard deviation; **: Mann-Whitney U test was used. Descriptive statistics were expressed as median (minimum-maximum); Bold p-values were considered statistically significant (p<0.05).

	Control (n=57)	Patient (n=60)	p-value
Somatosensory Amplification Scale - Total score	26.24 ± 8.48	31.15 ± 9.46	0.004*
Health Anxiety Inventory - Total score	13.0 [0.0–43.0]	18.50 [2.0–44.0]	<0.001**
Beck Anxiety Inventory - Total score	7.0 [0.0–34.0]	13.0 [0.0–62.0]	0.120**
				Beck Depression Inventory - Total score	5.0 [0.0–42.0]	11.0 [0.0–51.0]	0.002**

An analysis was made on whether gender, education level, disease severity, disease duration, BMI, and specific body area involvement were correlated with the SSAS, HAI, and BDI scores in the patient group, revealing a moderate correlation between specific body area involvement (especially the scalp and face) and SSAS score (Table 3). Bold p-values were considered statistically significant (p<0.05).

**Table 3:** Evaluation of the correlations between disease parameters and study scales *: Pearson’s correlation coefficient was used; Bold p-values were considered statistically significant (p<0.05); PASI: Psoriasis Area Severity Index; NAPSI: Nail Psoriasis Severity Index; SSAS: Somatosensory Amplification Scale; HAI: Health Anxiety Inventory; BDI: Beck Depression Inventory

Variables	Correlation	SSAS	HAI	BDI
	Coefficient			
PASI	r	-0.003	0.151	0.114
NAPSI	r	-0.152	0.092	0.019
Gender	r	0.002	-0.102	-0.064
BMI	r	0.078	0.068	0.086
Education level	r	0.012	0.063	-0.040
Disease duration	r	-0.122	0.141	0.028
Presence of psoriatic arthritis	r	0.044	-0.140	-0.092
Specific body area involvement				
Scalp	r	0.296*	0.214	0.081
Hands	r	-0.074	-0.015	-0.129
Face	r	0.289*	0.055	0.132
Nails	r	-0.002	-0.117	-0.001
Genital	r	-0.034	-0.050	-0.011

## Discussion

The present study found the mean total SSAS score and the median HAI score to be significantly higher in psoriasis patients than in the control group. The median BAI score was not statistically significantly different between the two groups although the median BAI score was higher in the patient group. The median BDI score was significantly higher in the patient group than in the healthy control group, and there was a moderate correlation between specific body area involvement (especially the scalp and face) and SSAS score.

A recent study in the United Kingdom assessed the magnitude of the mental health burden linked to skin diseases and reported that 93% of the patients stated that their self-esteem had been affected by their skin condition. As a result of such feelings about personal appearance and/or perceived stigma, many patients adopt avoidance and concealment as coping strategies, leading to social isolation [15]. Psoriasis, a life-long disease involving remission and relapse, significantly affects the quality of life. The psychosocial comorbidities that have been strongly linked to psoriasis include anxiety, depression, suicidal ideation, and substance misuse [16]. There have been no studies to date identifying a significant relationship between PASI score, indicating the clinical severity of psoriasis, and quality of life and daily functioning, although patients with psoriatic lesions in visible body areas have a lower quality of life scores [17]. Our study found no correlation between the PASI score and study scales. The involvement of visible body areas, such as the scalp and face, was significantly correlated with the SSAS score, which suggests that psoriasis can result in psychosocial effects, regardless of the severity of the disease. Depending on the scales used, depression affected 9-55% of patients with psoriasis and has a greater prevalence among those with more severe disease [18]. In contrast, the prevalence of anxiety among patients with psoriasis ranged from 7-48% and was not associated with disease severity [19]. Numerous studies have been conducted assessing depression and anxiety in patients with psoriasis. Kurd et al. analyzed the association of psoriasis with depression, anxiety, and suicidality in 146,042 patients with mild psoriasis, 3,956 patients with severe psoriasis, and 766,950 people without psoriasis [20]. As expected, those with psoriasis were found to be at increased risk of development of anxiety and depression, and the authors reported further that there were 10,400 cases of depression, 7,100 diagnoses of anxiety, and 305 cases of suicide attributable to psoriasis each year. Golpour et al. administered the BDI and the Spielberger State-Trait Anxiety Scale to 100 psoriasis patients and 100 healthy volunteers in a hospital-based case-control study and reported depression scores of 67% and 12% in the psoriatic patient and control groups, respectively [21]. According to the Spielberger State-Trait Anxiety Scale, 45% of the patients in the case group and 18% of the controls had anxiety. Similarly, a study from Italy reported that 62% of patients with psoriasis presented with depressive symptoms, and it was worthy of note that the prevalence of symptoms was higher in patients with a lower level of education [22]. In the present study, patients with psoriasis also recorded significantly higher depression scores, concurring with the literature.

Yılmaz et al. investigated the relationship between somatosensory amplification, anxiety, and depression among patients with hepatitis B and chronic diseases such as psoriasis. The SSAS scores and anxiety and depression levels were found to be significantly higher in patients with active hepatitis B infections than in those with inactive hepatitis B and the healthy controls. Based on this finding, the authors concluded that the routine check-up of patients with hepatitis B should include a psychiatric assessment [23]. A review evaluating the findings of 50 articles reported that the concept of somatosensory amplification represented a new approach to psychosomatic research that could better serve physicians in their efforts to gain an understanding of conditions in which the patient's psychiatric symptoms and clinical condition do not match. The approach also provides useful information steering the selection of the most appropriate pharmacological or psychological treatment. It is worth considering SSAS scores when treating patients with specific psychosomatic diseases (e.g. irritable bowel syndrome), chronic pain, psychiatric disorders (e.g. somatoform disorders), anxiety disorders and mood disorders, stress reaction (e.g. grief response and other major psychosocial events), infectious diseases, such as hepatitis B, and cardiac diseases [24].

This study has some limitations, including the small number of patients and the single-center, cross-sectional study design. Another limitation is that the scales used in the study were based on the patient's own reports.

## Conclusions

Patients with psoriasis score highly in depression, health anxiety, and somatosensory amplification, and there was a moderate correlation between specific body area involvement (especially the scalp and face) and SSAS score. The results of this study seem to indicate that psychiatric assessment and treatment approaches should be included in the treatment of such chronic skin diseases as psoriasis that follow a life-long remission and relapse pattern.

## References

[1] Yamanaka K, Yamamoto O, Honda T (2021). Pathophysiology of psoriasis: a review. J Dermatol.

[2] Nestle FO, Kaplan DH, Barker J (2009). Psoriasis. N Engl J Med.

[3] Honma M, Nozaki H (2021). Molecular pathogenesis of psoriasis and biomarkers reflecting disease activity. J Clin Med.

[4] Takeshita J, Grewal S, Langan SM, Mehta NN, Ogdie A, Van Voorhees AS, Gelfand JM (2017). Psoriasis and comorbid diseases: epidemiology. J Am Acad Dermatol.

[5] Cai Q, Teeple A, Wu B, Muser E (2019). Prevalence and economic burden of comorbid anxiety and depression among patients with moderate-to-severe psoriasis. J Med Econ.

[6] Cortés H, Rojas-Márquez M, Del Prado-Audelo ML, Reyes-Hernández OD, González-Del Carmen M, Leyva-Gómez G (2022). Alterations in mental health and quality of life in patients with skin disorders: a narrative review. Int J Dermatol.

[7] Barsky AJ, Goodson JD, Lane RS, Cleary PD (1988). The amplification of somatic symptoms. Psychosom Med.

[8] Lee S, Creed FH, Ma YL, Leung CM (2015). Somatic symptom burden and health anxiety in the population and their correlates. J Psychosom Res.

[9] Güleç H, Sayar K (2007). Reliability and validity of the Turkish form of the Somatosensory Amplification Scale. Psychiatry Clin Neurosci.

[10] Aydemir Ö, Kirpinar İ, Sati T, Uykur B, Cengisiz C (2013). Reliability and validity of the Turkish version of the Health Anxiety Inventory. Noro Psikiyatr Ars.

[11] Beck AT, Ward CH, Mendelson M, Mock J, Erbaugh J (1961). An inventory for measuring depression. Arch Gen Psychiatry.

[12] Hisli N (1989). A reliability and validity study of Beck Depression Inventory in a university student sample. J Turkish Psychol.

[13] Beck AT, Epstein N, Brown G, Steer RA (1988). An inventory for measuring clinical anxiety: psychometric properties. J Consult Clin Psychol.

[14] Ulusoy M, Sahin NH, Erkmen H (1998). Turkish version of the Beck Anxiety Inventory: psychometric properties. J Cogn Psychother.

[15] (2022). All-Party Parliamentary Group on Skin. Mental health and skin disease. https://www.appgs.co.uk/mental-health-and-skin-disease-report-2020/.

[16] Blackstone B, Patel R, Bewley A (2022). Molecular pathogenesis of psoriasis and biomarkers reflecting disease activity. Psoriasis (Auckl).

[17] Heydendael VM, de Borgie CA, Spuls PI, Bossuyt PM, Bos JD, de Rie MA (2004). The burden of psoriasis is not determined by disease severity only. J Investig Dermatol Symp Proc.

[18] Korman AM, Hill D, Alikhan A, Feldman SR (2016). Impact and management of depression in psoriasis patients. Expert Opin Pharmacother.

[19] Fleming P, Bai JW, Pratt M, Sibbald C, Lynde C, Gulliver WP (2017). The prevalence of anxiety in patients with psoriasis: a systematic review of observational studies and clinical trials. J Eur Acad Dermatol Venereol.

[20] Kurd SK, Troxel AB, Crits-Christoph P, Gelfand JM (2010). The risk of depression, anxiety, and suicidality in patients with psoriasis: a population-based cohort study. Arch Dermatol.

[21] Golpour M, Hosseini SH, Khademloo M, Ghasemi M, Ebadi A, Koohkan F, Shahmohammadi S (2012). Depression and anxiety disorders among patients with psoriasis: a hospital-based case-control study. Dermatol Res Pract.

[22] Esposito M, Saraceno R, Giunta A, Maccarone M, Chimenti S (2006). An Italian study on psoriasis and depression. Dermatology.

[23] Yilmaz A, Ucmak F, Dönmezdil S (2016). Somatosensory amplification, anxiety, and depression in patients with hepatitis B: impact on functionality. Medicine (Baltimore).

[24] Nakao M, Barsky AJ (2007). Clinical application of somatosensory amplification in psychosomatic medicine. Biopsychosoc Med.

